# Malaria epidemiology and interventions in Ethiopia from 2001 to 2016

**DOI:** 10.1186/s40249-018-0487-3

**Published:** 2018-11-05

**Authors:** Hiwot S Taffese, Elizabeth Hemming-Schroeder, Cristian Koepfli, Gezahegn Tesfaye, Ming-chieh Lee, James Kazura, Gui-Yun Yan, Guo-Fa Zhou

**Affiliations:** 1 National Malaria Program, Federal Ministry of Health, Addis Ababa, Ethiopia; 2 Program in Public Health, University of California, Irvine, CA 92697 USA; 3 Center for Global Health and Diseases, Case Western Reserve University, Cleveland, OH USA

**Keywords:** Malaria control, Policy, Ethiopia, *Plasmodium falciparum*, *Plasmodium vivax*, Epidemiology, Incidence, Spatial distribution

## Abstract

**Background:**

Ethiopia is one of the African countries where *Plasmodium falciparum* and *P. vivax* co-exist. Monitoring and evaluation of current malaria transmission status is an important component of malaria control as it is a measure of the success of ongoing interventions and guides the planning of future control and elimination efforts.

**Main text:**

We evaluated changes in malaria control policy in Ethiopia, and reviewed dynamics of country-wide confirmed and clinical malaria cases by *Plasmodium* species and reported deaths for all ages and less than five years from 2001 to 2016. Districts level annual parasite incidence was analysed to characterize the malaria transmission stratification as implemented by the Ministry of Health. We found that Ethiopia has experienced major changes from 2003 to 2005 and subsequent adjustment in malaria diagnosis, treatment and vector control policy. Malaria interventions have been intensified represented by the increased insecticide treated net (ITN) and indoor residual spraying (IRS) coverage, improved health services and improved malaria diagnosis. However, countrywide ITN and IRS coverages were low, with 64% ITN coverage in 2016 and IRS coverage of 92.5% in 2016 and only implemented in epidemic-prone areas of > 2500 m elevation. Clinical malaria incidence rate dropped from an average of 43.1 cases per 1000 population annually between 2001 and 2010 to 29.0 cases per 1000 population annually between 2011 and 2016. Malaria deaths decreased from 2.1 deaths per 100 000 people annually between 2001 and 2010 to 1.1 deaths per 100 000 people annually between 2011 to 2016. There was shrinkage in the malaria transmission map and high transmission is limited mainly to the western international border area. Proportion of *P. falciparum* malaria remained nearly unchanged from 2000 to 2016 indicating further efforts are needed to suppress transmission.

**Conclusions:**

Malaria morbidity and mortality have been significantly reduced in Ethiopia since 2001, however, malaria case incidence is still high, and there were major gaps between ITN ownership and compliance in malarious areas. Additional efforts are needed to target the high transmission area of western Ethiopia to sustain the achievements made to date.

**Electronic supplementary material:**

The online version of this article (10.1186/s40249-018-0487-3) contains supplementary material, which is available to authorized users.

## Multilingual abstracts

Please see Additional file 1 for translation of the abstract into the five official working languages of the United Nations.

## Background

Malaria morbidity and mortality have been significantly decreased in Ethiopia and worldwide in the past decade [1]. Ethiopia’s fight against malaria started many years ago and transmission of this infectious disease significantly decreased since 1959 [1, 2]. However, malaria still remains a major public health problem in Ethiopia [1]. Ethiopia has a population of nearly 100 million, and it is estimated that ~ 68% of the population is at risk of the disease [1, 3]. *Plasmodium falciparum* and *P. vivax* co-exist as major parasite species in Ethiopia [1]. This epidemiologic feature makes malaria control more complicated than in most African countries where *P. vivax* has low or nil endemicity.

Malaria transmission in Ethiopia occurs mainly at altitudes < 2000 m, although endemic regions > 2000 m have been reported [3–5]. The levels of malaria risk and transmission intensity, however, show marked seasonal, inter-annual and spatial variability, with the exception of the southwestern international border low land area where transmission is year-around [6–8]. In most regions of the country, the major transmission season is from September to December, following the main rainy season from June to September [3]. There is a short transmission season from April to May following the short rainy season in some regions [3]. *Anopheles arabiensis* is the predominant vector with *An. pharoensis*, *An. coustani*, *An. funestus* and *An. nili* having a minor role in transmission [9]. Generally, the diverse ecology of the country supports a wide range of transmission intensities ranging from low-seasonal to high-perennial transmission. For planning purposes and targeting of intervention strategies, the Federal Ministry of Health (FMoH) of Ethiopia has stratified the country’s malaria transmission burden using ‘woreda’ (district)-level transmission intensity according to annual parasite incidence per 1000 population (API) and elevation [3, 10]. Accordingly, four broad strata were identified by the mixed criteria of the FMoH and World Health Organization (WHO) — malaria free, low, moderate, and high transmission [3, 9].

*Plasmodium falciparum* are endemic in many regions of the country [3, 11–13]. *Plasmodium malariae* and *P. ovale* infection are uncommon and account for < 1% of confirmed malaria cases [3, 11]. Chloroquine (CQ) is currently the recommended first-line drug for treatment of vivax malaria [11]. In vivo monitoring of uncomplicated vivax malaria cases indicates that the CQ is generally efficacious; however, treatment failures have been reported [14–17]. The success of malaria control efforts has largely depended on financial support from donor funds [1].

In the past decade Ethiopia has made significant strides in expanding coverage of key malaria interventions throughout the country. Indoor residual spraying (IRS) using dichlorodiphenyltrichloroethane (DDT) was introduced in 1959 with the global malaria eradication campaign, and since then different chemical insecticides have been used for malaria control [9, 18, 19]. Insecticide-treated nets (ITN) were introduced in 1997 as an additional intervention [10]. Chloroquine was the first line treatment of all malaria species in Ethiopia before 1998. It was replaced by sulfadoxine-pyrimethamine (SP) after 1998 for the treatment of uncomplicated *P. falciparum* due to widespread decline in the efficacy of CQ [20, 21]. Parasites soon developed resistance to SP drugs [22, 23]. Planning for scaling-up malaria prevention and control interventions started in 2003 with the support from the Global Fund to Fight AIDS, Tuberculosis and Malaria (GFATM) [3]. In 2004, the FMoH introduced artemisinin-based combination therapy (ACT) as the first-line drug for treatment of *P. falciparum* malaria as well as rapid diagnostic tests (RDT) to improve diagnosis and long-lasting insecticidal nets (LLINs) as a method of preventing transmission of parasite from mosquitoes to people [3]. Major scale-up began in 2005 with country-wide distribution of RDTs, ACTs, LLINs and implementation of IRS [3].

The aim of this study was to provide comprehensive evaluation of changes in national malaria control policy and progress made in malaria control interventions and malaria epidemiology in Ethiopia. We discuss obstacles to malaria elimination and review national malaria data from 2000 to 2016 and analyse these data in order to advance understanding of how changes in policy have impacted temporal changes in malaria transmission in the country.

## Main text

### Data collection

Changes in malaria control policy were reviewed based on different sources including FMoH publications on malaria diagnosis, treatment and vector control guidelines, WHO guidelines, the President’s Malaria Initiative (PMI), and Ethiopian malaria control operational plans and reports [10, 11, 18, 19, 24–30]. National malaria data from health facilities were collected through the Health Management Information System (HMIS). Total confirmed and clinical malaria cases, malaria cases by species, and reported deaths for all ages and age < 5 years between 2001 and 2016 were reviewed. Incidence rate was expressed as malaria cases/1000 people/year and death rate as deaths/100000 people/year. Changes in number of health facilities, annual number of tests (microscopic examination of blood smears and RDT) of suspected malaria cases, reports on new insecticide treated nets (ITN and LLIN), and IRS coverage from 2007 to 2016 were obtained from the FMoH. The interventions were targeted to suit local epidemiological situations, with case management being strengthened in all areas. LLIN and IRS were implemented in targeted areas before 2011 [3].

Annual parasite incidence (API) according to district was analysed to characterize the district as malaria-free (API < 1.0), low (1.1–5.0), moderate (5.1–100), or high transmission (> 100) and to map transmission dynamics according to district [10]. Malaria records at the district level nation-wide were only available since 2013, and annual district level micro-plans were used to determine API of each district in 2013 and 2016 (with the exception of the Somali region where information from the National Public Health Emergency Management Plan was used). Data from a total of 845 districts were analysed.

### Statistical analysis

API and malaria death rates were calculated based on 2007 Ethiopian government census populations and 2013 projections at district and national levels assuming constant population growth rate over the study period. Percent changes in malaria indicators from 2013 to 2016 were calculated according to the formula: (Value2013–Value2016)/Value2013 × 100. Maps of API at district level were generated using ArcGIS 10.0 (ESRI, Redlands, CA, USA). Numbers of districts with malaria-free, low, moderate and high API were calculated separately for 2013 and 2016. The total population and percentage composition in different transmission categories was calculated accordingly. The number of districts and total populations in different transmission categories were compared between 2013 and 2016 using *χ*^2^-test through free online statistical tool VassarStats: Website for Statistical Computation (http://vassarstats.net/index.html).

### Evolving malaria control policy

**Fig. 1:**
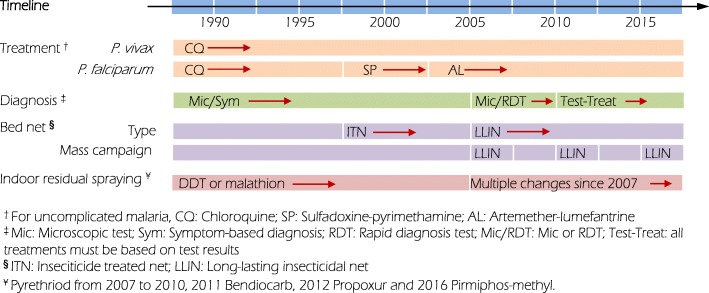
Malaria intervention scheme in Ethiopia from 1990 to 2016

The major policy changes in treatment were the replacement of CQ with SP for the first line treatment of uncomplicated *P. falciparum* malaria in 1998 and subsequent replacement with artemether-lumefantrine (AL), an ACT drug, in 2004 (Fig. 1). Also in 2004, RDTs were implemented in Ethiopia to improve diagnosis; however, the test-treat policy, i.e., administration of antimalarial drugs was based on test results, was implemented in 2010 (Fig. 1). According to current national guidelines, laboratory diagnosis is based on microscopic examination of blood smears at hospitals and health centres. RDTs are used at health posts and by health extension workers based in local communities.

CQ remains the first-line treatment of uncomplicated vivax malaria (Fig. 1) [14–17]. Anti-relapse therapy with primaquine for *P. vivax* malaria is currently recommended, with supervised use, in malaria elimination-designated districts [11].

LLIN were introduced into Ethiopia in 2004 as a method of malaria prevention and control [3]. Major scale-up began in 2005 with country-wide distribution of LLINs free of charge (Fig. 1) [3]. LLIN distribution campaigns were carried out in 2005, 2010 and 2015. Before 2011 LLIN coverage targeted all age groups in endemic areas below 2000 m altitude. Long lasting insecticidal nets have been provided free in all malaria endemic areas since 2011 [27–29].

DDT and malathion were the main insecticides used for IRS before 2007 (Fig. 1) [9]. Due to concerns of high-level resistance to and long-term field residual effect of DDT, pyrethriods were used for IRS from 2007 to 2010. Bendiocarb and propoxur were used in different districts in 2011 and 2012, respectively. Pirmiphos-methyl was introduced in PMI spraying districts in 2016 (Fig. 1) [9, 31]. Indoor residual spraying was used in epidemic-prone areas situated at altitudes of 2000 to 2500 m. [3]. New guidelines promote selectively IRS intervention based on API, i.e., IRS is used in both low (API 1~ 5) and high transmission areas (API ≥ 100). IRS is also used in low-risk populations located in highland epidemic fringe areas [25, 28], however, this new recommendation has not been implemented in the field at the time this study was conducted.

Larval source reduction and laviciding has been recommended as part of the vector control measures [11, 32]. Larval control is recommended for all malaria affected areas where breeding sites are permanent, identifiable, and few [25, 30]. Temephos (Abate®) is the recommended larvicide [11].

### Changes in clinical malaria incidence

**Fig. 2:**
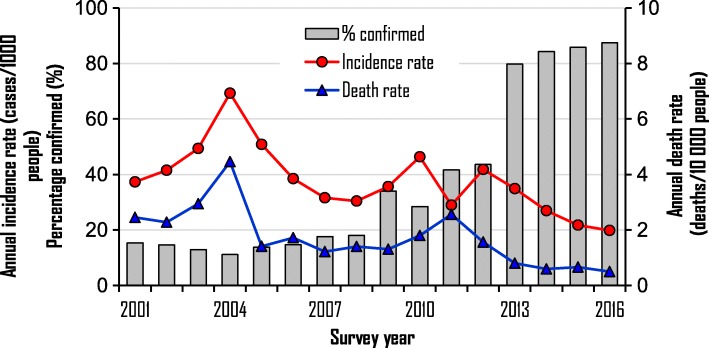
National level annual total reported malaria cases, deaths due to malaria, and proportion of cases confirmed by microscopy or RDT from 2001 to 2016

There has been an overall reduction in reported malaria incidence and deaths, increases LLIN and IRS coverage and the number of health facilities from 2000 to 2016 (Fig. 2 and Table 1). Population pooled microscopically or RDT confirmed cases increased from < 20% before 2008 to ~ 40% by 2012 and > 80% since 2013 (Fig. 2). There were three small malaria case increases in 2004, 2010, and 2012 (Fig. 2). Malaria incidence showed a general declining trend from 2001 to 2016 with some annual variations (Fig. 2). The average annual incidence declined from 43.1 cases per 1000 people annually between 2001 and 2010 (before LLINs were made available free to all malaria affected people) to 29.0 cases per 1000 people between 2011 and 2016. This declining trend was more pronounced from 2014 to 2016 (after two rounds of mass LLIN distribution) when the average annual incidence was 22.9 cases per 1000 people (Fig. 2). Malaria death incidence showed a similar declining trend (Fig. 2). There were 2.1 deaths per 100 000 people annually between 2001 and 2010 and 1.1 cases per 1 000 000 people annually between 2011 and 2016, a drop of 47%. The declining trend was more pronounced from 2014 to 2016, with an annual incidence of 0.6 cases per 100 000 people (Fig. 2, Table 1).

**Table 1:** Changes in malaria epidemiological indicators in Ethiopia from 2013 to 2016

Year	2013	2016	Change (%)
Total reported cases			
	3 316 013	1 962 996	−40.8
Confirmed	2 645 454	1 718 504	−35.0
(% confirmed)	79.8	87.5	9.7
Incidence rate (cases/1000 population)	36.6	20.2	− 44.8
*P. falciparum* proportion (%)	63.8	66.5	4.2
Age			
< 5 years	785 895	319 795	− 59.3
(% of total)	23.7	17.1	−27.8
≥ 5 years	2 530 118	1 553 231	−38.6
Malaria admission			
< 5 years	6602	6766	2.5
(% of total)	16.5	16.9	2.5
≥ 5 years	33 525	33 345	−0.5
Malaria deaths			
< 5 years	43	12	−72.1
(% of total)	5.6	2.4	−58.2
≥ 5 years	721	498	−30.9

### Malaria diagnosis and parasite species

**Fig. 3:**
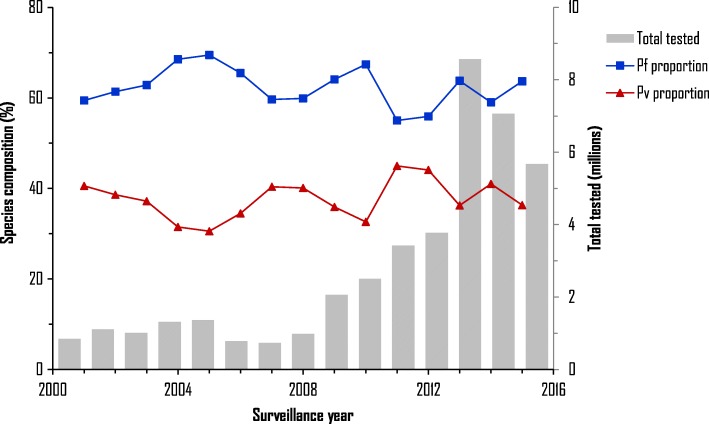
National level annual total number of suspected cases tested and parasite species composition from 2001 to 2016. Pf: *Plasmodium falciparum*; Pv: *Plasmodium vivax*

The number of RDT and microscopy tested suspected malaria cases increased significantly from below one million before 2008 to 8.6 million in 2014 and an average of 7.1 million from 2014 to 2016 (Fig. 3). Case confirmation rate by microscopy and/or RDT increased from 79.8% in 2013 to 87.5% in 2016 (Table 1). Although there was year-to-year variation, malaria species changed little over time. *Plasmodium falciparum* accounted for ~ 60% of cases (range 55–69%) and *P. vivax* 40% (range 31–45%) from 2001 to 2016 (Fig. 3, Table 1).

### Intensification of malaria interventions and increase in number of health facilities

**Fig. 4:**
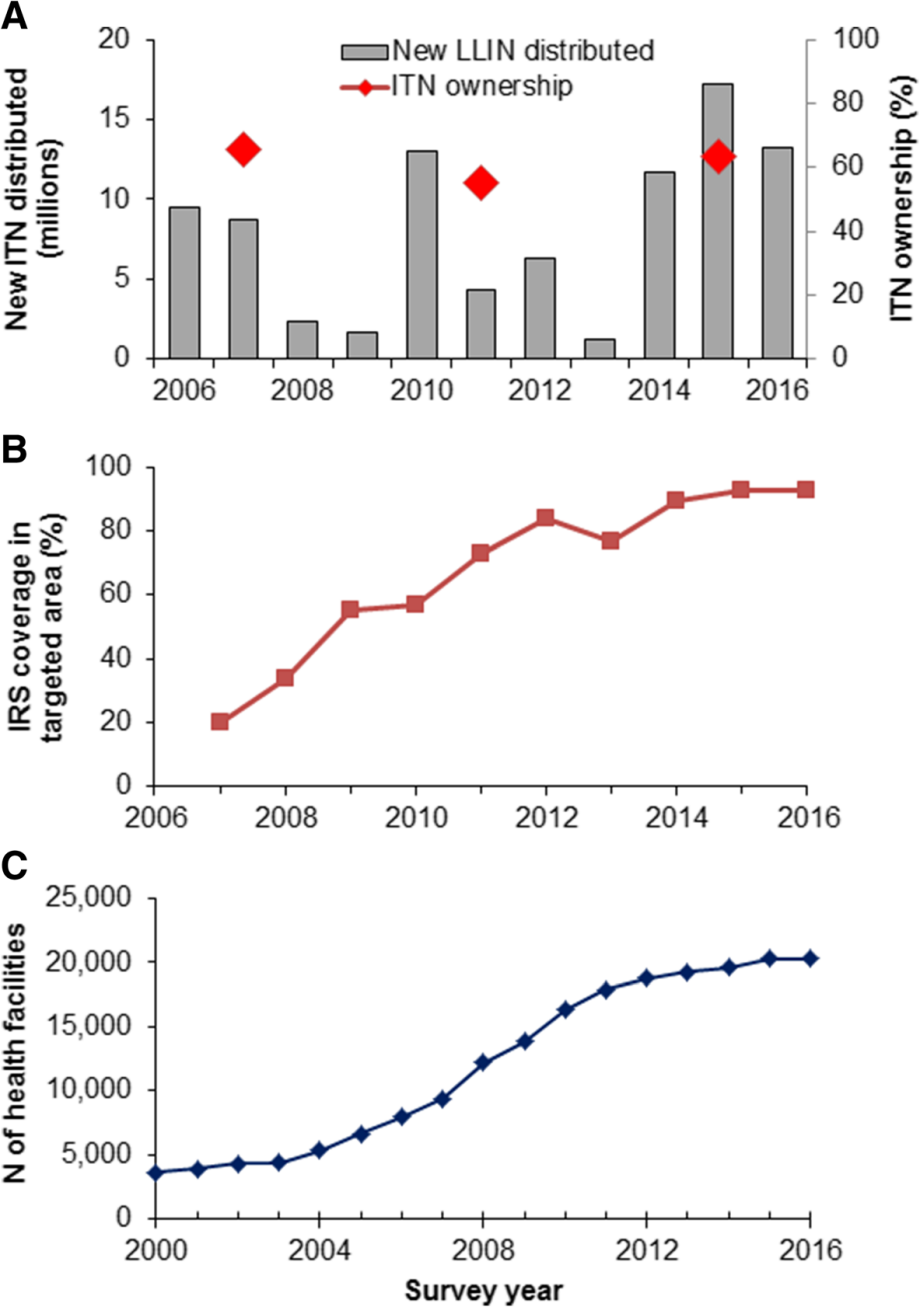
National level: **a**. Annual total number of new LLIN distributed from 2006 to 2016 and household LLIN ownership (% of households with at least one LLIN) in 2007, 2011 and 2015; **b**. IRS coverage (%) in targeted areas, and **c**. Total number of health facilities in Ethiopia from 2000 to 2016. Pf: *Plasmodium falciparum*; Pv: *Plasmodium vivax*; LLIN: long-lasting insecticidal net; and IRS: indoor residual spraying

FMoH has distributed 18.2 million ITN/LLINs between 2006 and 2007, 13.0 million LLINs in 2010 and 42.4 million LLINs between 2014 and 2016 (Fig. 4a). However, the nation-wide LLIN ownership (proportion of households with at least one LLIN) has been relatively low, 65% in 2007, 55% in 2011 and 64% in 2015. IRS coverage increased from 20.0% in 2007 to 92.5% in 2016 in epidemic-prone areas located at altitudes ≥2500 m (Fig. 4b). IRS was not implemented in other non-targeted areas. Meanwhile, the number of health facilities nationally has steadily increased from 3612 in 2000 to 6604 in 2005, just before the scale-up of malaria interventions. The total number of health facilities in 2016 was 20 283 (Fig. 4c).

### Changes in spatial transmission patterns

Overall malaria-related hospital admissions showed no significant change between 2013 and 2016 (Table 1). However, malaria deaths decreased significantly from 2013 to 2016. Reported malaria cases in children < 5 years decreased by 59.3% between 2013 and 2016, from a total of 785 895 to 319 795, respectively (Table 1). Malaria deaths dropped by 72.1% over the same time interval, only 12 deaths were reported in 2016 (Table 1).

**Fig. 5:**
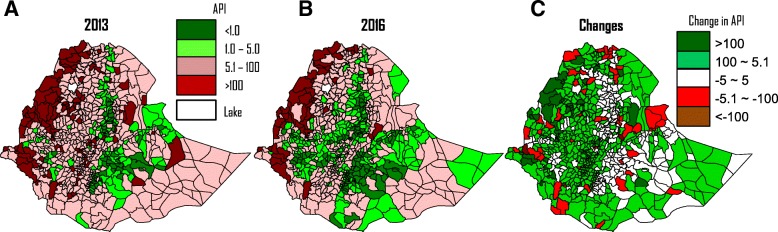
Malaria transmission intensity maps by districts in **a**: 2013, **b**): 2016, and **c**): Difference in API between 2013 and 2016 (positive indicating decrease and negative indicating increase). API: annual parasite incidence per 1000 population

Spatially, areas of moderate to high transmission areas decreased between 2013 and 2016 (Fig. 5a and b). Areas with API > 100 (high transmission) significantly shrank whereas areas with API ≤5 (low transmission) significantly increased, especially in the central west region of Ethiopia where high transmission totally disappeared (Fig. 5, Table 2). Overall, the number of high transmission districts dropped from 14.3 to 6.4% whereas low transmission districts increased from 22.5 to 37.4% (*χ*^2^ = 59.97, degree of freedom = 3, *P* < 0.0001) (Table 2). Proportion of the population in high risk areas decreased from 9.5% in 2013 to 3.3% in 2016 while the proportion in low risk areas increased from 26.7 to 42.7% (*χ*^2^ = 3.84, *df* = 3, *P* = 0.0501) (Table 2).

**Table 2:** Changes in distribution of malaria transmission intensity and affected populations at district level in Ethiopia from 2013 to 2016

API	Number of districts				Change in percentage^a^	Total population (%)				Change in percentage^a^
	2013	%	2016	%		2013	%	2016	%	
< 1.0	60	7.2	92	10.9	3.7	7 887 232	9.2	11 730 861	12.7	3.4
1.0–5.0	128	15.3	224	26.5	11.2	14 941 965	17.5	27 787 000	30.0	12.5
5.1–100	527	63.2	475	56.2	(7.0)	54 487 279	63.8	50 005 687	54.0	(9.8)
> 100	119	14.3	54	6.4	(7.9)	8 133 374	9.5	3 093 995	3.3	(6.2)
Total	834		845			85 449 850		92 617 543		

^a^Difference in percentages from 2013 to 2016, numbers in brackets indicating negative

The reduction in clinical malaria was not uniform across the country (Fig. 5c). The majority of districts experienced reductions in incidence rates from 2013 to 2016 (Fig. 5c), with two districts having reductions of API > 500, i.e., reduction in API of 781 in Masha Woreda, Sheka Zone of SNNPR region and 641 in Fagta Lakoma Woreda, Awi Zone of Amhara Region. By contrast, there were 59 districts with noticeable increases in clinical malaria incidence (API > 5) from 2013 to 2016, and two districts with increases in API > 100, i.e., 131 in Awash Fentale Woreda, Zone 3 of Afar Region, and 118 in Itang Woreda, Agnuak Zone of Gambella Region.

### Progress has been made and major challenges

#### Progress has been made and future new interventions

Ethiopia is one of the few countries in sub-Sahara Africa with a policy to provide the main malaria prevention and control services free of charge, including malaria diagnosis and treatment, mosquito nets and IRS [3, 11]. This policy has ensured accessibility of the poor to malaria interventions and, hence, protection from malaria to increase household economic productivity. We emphasize that, in Ethiopia, the success of these policies largely depended on donor funds [1, 25–30]. The overall reduction of malaria burden is evident since 2013, reflected in the shrinkage of the malaria map between 2013 and 2016, particularly in the central highlands of Ethiopia where no high transmission was observed in 2016. Anti-relapse therapy with primaquine for *P. vivax* malaria is not currently recommended in malaria-endemic areas [1]. However, the national malaria case management guidelines outline the specific recommendations, including the use of a full 14-day course of primaquine for radical cure of *P. vivax* at malaria elimination-designated districts and single dose primaquine for gametocytocidal activity against *P. falciparum* in all areas of the country [26, 30]. With this new intervention, the case burden is expected to decrease in the near future.

There was clear decrease in malaria incidence rate and malaria death rate in Ethiopia from 2001 to 2016; however, malaria case number and incidence remained high—19.8 cases per 1000 people reported in 2016 exceed the WHO standard for “pre-elimination.” Overall LLIN ownership and IRS coverage also continue to be low. In 2016, LLIN ownership rate was 62% nation-wide, which is far lower than its neighbouring country of Kenya [32, 33]. IRS coverage was even lower, nation-wide average coverage of 23% from 2014 to 2016 [32]. Therefore, there is ample opportunity to increase LLIN and IRS coverage which would be expected to decrease malaria transmission further. There is a clear need to improve the mechanism of LLIN distribution so that all at risk populations can be adequately covered.

#### New risk factors

Although the FMoH of Ethiopia has classified areas at altitudes > 2000 m as malaria-free zones [10], they are not entirely malaria free at the district level. In fact, many of these districts have moderate APIs according to this study. These areas may have scattered seasonal micro-geographic local transmissions due to local environmental suitability [10, 34]. For example, construction of dams might increase malaria transmission in the valleys of highland areas where overall malaria transmission was very low [35]. These dams create transmission hot spots along valleys where irrigation is important for farming [36]. Population migration and treatment-seeking behaviour may have contributed to the high level of API observed in these highlands [37]. Further investigation of this issue is warranted.

#### Confirmed cases are still high

There have been significant advances in malaria diagnosis and prevention in Ethiopia since the beginning of scale up in 2004 [8, 28, 38, 39]. Before 2008, over 80% of the reported cases were presumptive cases lacking parasitological confirmation. Presumed treatment has declined greatly in the last few years. For example, in 2016, the annual performance report showed that 6 367 309 suspected cases were examined by microscopy or RDTs, with a total number of 1 718 504 confirmed cases by either method. Meanwhile, the FMoH reported the total number of clinical malaria cases to be 1 962 996, and the proportion of clinically diagnosed cases based on symptoms alone reduced to 12.5% in 2016. This was a result of successful implementation of the malaria test-treat policy.

#### Challenges for further reducing malaria transmission

Malaria transmission is very heterogeneous across Ethiopia. The malaria burden is still high in western Ethiopia near the borders of Sudan and South Sudan. This suggests that these high burden areas need to be prioritized to sustain the gains made so far and achieve malaria elimination. In central Ethiopia, particularly the central highlands, malaria transmission has been significantly reduced, and many of these areas have reached pre-elimination levels of transmission. These results are similar to those of a previous study showing a reduction in malaria incidence in central Ethiopia after ITN distribution [40–42]. Nevertheless, malaria epidemiology in western Ethiopia has clearly been under-studied [10, 38, 39]. In addition, dealing with how to prevent resurgence and re-introduction is a new challenge for the central highlands. Finally, in the Somalia Region of eastern Ethiopia bordering Somalia where malaria reporting is less accurate due to inadequate capacity, better surveillance systems are desperately needed in the era of malaria elimination [10].

## Conclusions

Malaria morbidity and mortality have significantly declined in Ethiopia since 2000. However, transmission remains high in the western border area near Sudan and South Sudan. Appropriate vector control methods that target outdoor transmission are needed, especially for migrant workers and for people who work at night. Additional efforts are needed to target the high transmission area of western Ethiopia to sustain achievements made to date and hasten the endeavour of achieving malaria elimination.

## Additional file

Additional file 1: Multilingual abstracts in the five official working languages of the United Nations. (PDF 423 kb)

## Abbreviations

ACT: Artemisinin-based combination therapy
AL: Artemether-lumefantrine
API: Annual parasite incidence
CQ: Chloroquine
FMoH: Federal Ministry of Health
GFATM: Global Fund to Fight AIDS, Tuberculosis and Malaria
HEW: Health Extension Workers
HMIS: Health Management Information System
IRS: Indoor residual spraying
ITN: Insecticide-treated net
LLIN: Long-lasting insecticidal net
Pf: *Plasmodium falciparum*
PMI: President’s Malaria Initiative
Pv: *Plasmodium vivax*
RDT: Rapid diagnosis test
SNNPR: Southern Nations, Nationalities, and Peoples’ Region, Ethiopia
SP: Sulfadoxine-pyrimethamine
WHO: World Health Organization
