# Supplementary material for: Malaria epidemiology and interventions in Ethiopia from 2001 to 2016
Source: Infect Dis Poverty. 2018 Nov 5;7:103. doi: 10.1186/s40249-018-0487-3 (PMC6217769; doi:10.1186/s40249-018-0487-3)
Supplement: Supplementary file 1 — Multilingual abstracts in the five official working languages of the United Nations. (PDF 423 kb) [file 40249_2018_487_MOESM1_ESM.pdf]

Translation of the abstract into the five official working languages of the United Nations

وباء الملاريا والتدخلات بدولة إثيوبيا من 2001 حتى 2016

Ming- ,Hiwot S. Taffese, Elizabeth Hemming-Schroeder, Cristian Koepfli, Gezahegn Tesfaye[1]
chieh Lee, James Kazura, Guiyun Yan and Guofa Zhou

الملخص

الخلفية: تُعدّ إثيوبيا أحدى الدول الأفريقية التي يتعايش فيها كلا من الطفلييّنالبلازمود المنجلي والبلازمود النشيط معًا وتعتبر مراقبة الحالة الجارية لانتشار الملاريا وتقيمهما مكّونان مهمان لمكافحة الملاريا، فهما يمثلان مقياس لنجاح التدخلات السارية وارشادات لتخطيط الجهود المستقبلية الرامية لمكافحة الملاريا والقضاء عليها.

النص الرئيس: نُقيّم التغيّرات الناجمة في سياسة مكافحة الملاريا بإثيوبيا مُنذ 1990 حتى 2016، كما نُراجع ديناميكية حالات الملاريا الإكلينيكية المؤكدة على نطاق الدولة التي أصيبت عن طريق أحد أنواع طفيل البلازمود، وتم الإبلاغ بالوفيات لكل الأعمار، لمدة لا تقل عن خمس سنوات من 2001 حتى 2016. تم تحليل مدى الإصابة السنوية بالطفيل على مستوى المقاطعات إلى أنماط لتقسيم انتشار الملاريا كما هومطبق من خلال وزارة الصحة. وجدنا أن إثيوبيا شهدت تغيّرات كبيرة من 2003 حتى 2005، وما لحقه من تعديل في تشخيص الملاريا وعلاجها وسياسة مكافحة ناقلتها. تضاعفت قوة التدخلات ضد للملاريا بالتوسع في استخدام الناموسيات المعالجة بمبيد حشري (ITN)، وتوسيع نطاق الرش الموضعي للأماكن المغلقة (IRS)، و تعزيز الخدمات الصحية، وتحسين تشخيص الملاريا. وعلى الرغم من ذلك، تُغطي الناموسيات المعالجة بمبيدات الحشرات والرش الموضعي للأماكن المغلقة مساحة صغيرة، فكانت نسبة تلك التغطية في العام 2016 فيما يخص (ITN) مقدار 64%، بينما نسبة 92.5% فيما يخص (IRS) في العام 2016، ولم يتم تطبيقها سوى في المناطق المعرضة للأوبئة بمساحة بالغة >2500 ميل. انخفض معدل انتشار الملاريا اكلينيكيًا من متوسط 43.1 حالة لكل 1000 شخص من السكان سنويًا بين الأعوام 2001-2010 إلى 29.0 حالة لكل 1000 شخص من السكان بين الأعوام 2011-2016، كما تناقصت حالات وفيات الملاريا من 2.1 حالة لكل 100000 شخص سنويًا بين الأعوام 2001-2010 إلى 1.1 حالة لكل 100000 شخص سنويًا بين الأعوام 2011-2016. وهناك تراجع في خريطة انتشار الملاريا، وانحسرت معدلات العدوى المرتفعة بصفة رئيسة في منطقة الحدود الدولية الغربية. ظلت نسبة طفيل البلازمود المنجلي الحامل للملاريا دون تغير تقريبًا في الفترة من 2000 حتى 2016، مما يشير إلى أنه ينبغي بذل المزيد من الجهود للقضاء على الانتشار.

الاستنتاج : انخفضت حالات الاصابة بالملاريا وحالات الوفاه انخفاضًا كبير في إثيوبيا مُنذ 2001، غير أنَّه لا تزال حالات الإصابة في ارتفاع، كما توجد فجوات ضخمة بين مُلاك الناموسيات المعالجة بمبيد حشري (ITN) وبين الامتثال لتطبيقها في المناطق الموبوءة بالملاريا. ومن الضروري بذل جهود إضافية لاستهداف مناطق العدوى المرتفعة شرق إثيوبيا، للحفاظ على ما تحقق من إنجازات حتى الآن.

Translated from English version into Arabic by Mohamed Fouad and Abed Shawky, through

TRANSLATORS
WITHOUT BORDERS

埃塞俄比亚疟疾的流行病学和防治: 2001–2016

Hiwot S. Taffese, Elizabeth Hemming-Schroeder, Cristian Koepfli, Gezahegn Tesfaye, Ming-chieh Lee, James Kazura, Guiyun Yan and Guofa Zhou

摘要

引言：埃塞俄比亚是非洲仅有的恶性疟和间日疟并存的几个国家之一。监测和评价目前的疟

疾病流行状况是疟疾病防治的重要组成部分，它不仅是评估当前实施的防治措施是否成功，同时为制定下一步的防治乃至消灭疟疾病的措施提供可靠依据。
正文：我们评估了从 1990 到 2016 年期间埃塞俄比亚政府疟疾病防治政策的变化，分析了自 2001 到 2016 年期间埃塞俄比亚全国确诊的疟疾病病例变化动态，疟原虫种类，报告的全部疟疾死亡病例和 5 岁以下儿童疟疾的死亡病例。根据埃塞俄比亚卫生部的疟疾病流行程度分类标准，我们分析了以地区为单位的不同流行区的疟疾病分布特征及其变化。我们发现埃塞俄比亚的疟疾病防治政策经历了 2003-2005 年的主要转变和后续的疟疾病诊断手段，疟疾病治疗和蚊媒防治策略的连续调整。疟疾病防治手段明显加强了，作为主要代表的目前的防治措施包括长效农药处理的蚊帐，室内农药喷洒，逐步改进的医疗服务系统和有效的疟疾诊断手段。但是，全国范围的蚊帐使用率和室内农药喷洒比例还很低, 2016 年的蚊帐覆盖率是 64%，室内农药喷洒比例是 92.5%，但是室内农药喷洒只限制在海拔 2500 米以上的低流行易于爆发的地区。疟疾病发病率下降到从 2001-2010 年期间的每千人每年 43.1 例到 2011-2016 年期间的 29.0 例；疟疾病死亡率下降到从 2001-2010 年间的每十万人每年 2.1 例到 2011-2016 年期间的 1.1 例。疟疾病流行范围逐步缩小，高发区逐步缩小到西部国际边境地区。但是恶性疟的比例从 2000 到 2016 年期间基本没有减少，表明需要进一步加强防治。
结论：自 2001 年以来，埃塞俄比亚疟疾病的发病率和死亡率明显降低了，但是全国范围的疟疾病病例还很高，在疟疾流行区存在蚊帐使用率低的问题。同时需要进一步加强西北地区疟疾高发区的防治，以保持目前取得的防治成果防治疟疾病的复发。

Translated from English version into Chinese by Guo-Fa Zhou

Épidémiologie du paludisme et interventions en Éthiopie de 2001 à 2016

Hiwot S. Taffese, Elizabeth Hemming-Schroeder, Cristian Koepfli, Gezahegn Tesfaye[1], Ming-chieh Lee, James Kazura, Guiyun Yan et Guofa Zhou

Résumé

Contexte : L'Éthiopie fait partie des pays africains dans lesquels coexistent le *Plasmodium falciparum* et le *P. vivax*. La surveillance et l'évaluation de l'état actuel de la transmission du paludisme constituent un aspect important du contrôle de la maladie, qui permet de mesurer le succès des interventions en cours et de mieux planifier les contrôles à venir et les efforts d'élimination.
Corps du texte : Nous avons évalué les changements opérés dans les programmes de contrôle du paludisme en Éthiopie de 1990 à 2016, examiné les dynamiques des cas confirmés et à manifestations cliniques de paludisme à *Plasmodium* à l'échelle nationale et signalé les décès à tout âge et chez les enfants de moins de 5 ans entre 2001 et 2016. L'incidence parasitaire annuelle à l'échelle des districts a été analysée dans le but de définir la stratification de la transmission du paludisme, telle qu'elle est mise en œuvre par le Ministère de la Santé. Nous avons constaté que l'Éthiopie avait connu des changements majeurs de 2003 à 2005 et qu'elle avait ensuite ajusté ses politiques de diagnostic, de traitement et de lutte antivectorielle en matière de paludisme. Les interventions contre le paludisme se sont intensifiées, comme en témoignent l'augmentation de l'utilisation de moustiquaires imprégnées d'insecticide et des pulvérisations intradomiciliaires d'insecticide à effet rémanent ainsi que l'amélioration des services de santé et du diagnostic du

paludisme. Néanmoins, l'utilisation de moustiquaires imprégnées d'insecticides et de pulvérisations intradomiciliaires d'insecticide à effet rémanent à l'échelle nationale reste basse, la couverture se limitant à 64 % pour les moustiquaires et à 92,5 % pour les pulvérisations en 2016, ces deux méthodes n'étant mises en oeuvre que dans les zones à risque situées à plus de 2 500 mètres d'altitude. Le taux d'incidence clinique du paludisme est passé de 43,1 cas par an entre 2001 et 2010 à 29,0 pour 1000 habitants par an entre 2011 et 2016. Le nombre de décès dus au paludisme est passé de 2,1 décès pour 100 000 personnes par an entre 2001 et 2010 à 1,1 décès par an pour 100 000 personnes entre 2011 et 2016. On observe une diminution de la carte de transmission du paludisme et la transmission élevée se limite principalement à la zone frontalière internationale occidentale. La proportion de paludisme*P. falciparum* n'a pratiquement pas évolué entre 2000 et 2016, ce qui indique que des efforts supplémentaires sont nécessaires pour supprimer la transmission.
Conclusions: La morbidité et la mortalité dues au paludisme ont été considérablement réduites en Éthiopie depuis 2001. Cependant, l'incidence du paludisme est encore élevée et il existe des écarts importants entre l'appropriation des moustiquaires imprégnées d'insecticide et la conformité dans les zones touchées par le paludisme. Des efforts supplémentaires sont nécessaires pour cibler la zone de transmission élevée de l'ouest de l'Éthiopie si l'on veut maintenir les progrès accomplis à ce jour.

Translated from English version into French by Isabelle Redon and Dorothée ALTIMIRA, through

TRANSLATORS
WITHOUT BORDERS

Эпидемиология и мероприятия по борьбе с малярией в Эфиопии с 2001 по 2016 год

Хивот С. Таффесе, Элизабет Хемминг-Шредер, Кристиан Кепфли, Гезахейн Тесфайе[1], Минчиэ Ли, Джеймс Казура, Гуйюнь Янь и Гофа Чжоу

Аннотация

История вопроса: Эфиопия является одной из африканских стран, в которых сосуществуют *Plasmodium falciparum* и *Plasmodium vivax* . Наблюдение и оценка текущего состояния передачи малярии являются важными составляющими борьбе с данным заболеванием, так как они используются для измерения успеха постоянных вмешательств, а также направляют планирование будущего контроля и мероприятий по его ликвидации.
Основной текст: Мы произвели оценку изменений в политике по борьбе с малярией в Эфиопии с 1990 по 2016 год, а также сделали обзор какк повсеместных подтверждённых и клинических случаев данного заболевания, вызванных разными видами *плазмодия* , так и зарегистрированных смертей по всем возрастным группам за период менее пяти лет с 20012 по 2016 год. Чтобы охарактеризовать стратификацию передачи малярии, произведённую Министерством здравоохранения, была проанализирована ежегодная распространённость заражения паразитами на районном уровне. Мы обнаружили, что в Эфиопии с 2003 по 2005 год произошли значительные изменения и последующая корректировка политики в области диагностики, лечения и борьбы с переносчиками малярии. Мероприятия по борьбе с

малярией были усилены в связи с увеличением охвата сетками, обработанными инсектицидом длительного действия (СОИДД), а также вследствие обработки помещений инсектицидами остаточного действия (ОПИОД) одновременно с улучшением медицинского обслуживания и диагностики малярии. Тем не менее охват СОИДД и ОПИОД в масштабе страны был низким: в 2016 году охват СОИДД составлял 64%, а охват ОПИОД составлял 92,5% и осуществлялся только в районах, находящихся на уровне ниже 2500 метров над уровнем моря и предрасположенных к эпидемиям. Уровень заболеваемости клинической малярией снизился со среднего уровня в 43,1 случаев на 1000 человек в год в период с 2001 по 2010 год до 29,0 случаев на 1000 жителей в год в период между 2011-2016 годами, а смертность от малярии уменьшилась с 2,1 смертей на 100 000 человек ежегодно в период с 2001 по 2010 год до 1,1 смерти на 100 000 человек ежегодно в период с 2011 по 2016 год. Наблюдалось сокращение карты передачи малярии, а высокий уровень передачи ограничивался главным образом западным районом в непосредственной близости к международной границе. Пропорция малярии, вызванной *Plasmodium falciparum* , оставалась относительно неизменной в период с 2000 по 2016 год, что указывает на необходимость дальнейших усилий по подавлению передачи заболевания.

Выводы: Заболеваемость малярией и смертность от малярии в Эфиопии значительно сократились с 2001 года, однако заболеваемость малярией по-прежнему высока, а также имели место значительные несоотвествия между наличием СОИДД и соблюдением правил в малярийных районах. Для решения проблемы высокого уровня передачи малярии в западных районах Эфиопии, а также для сохранения уже достигнутого на сегодняшний день необходимы дополнительные усилия.

Translated from English version into Russian by Ira Kulinevych and Liudmila Tomanek, through

TRANSLATORS
WITHOUT BORDERS

Epidemiología e intervenciones contra la malaria en Etiopía de 2001 a 2016

Hiwot S. Taffese, Elizabeth Hemming-Schroeder, Cristian Koepfli, Gezahegn Tesfaye[1], Ming-chieh Lee, James Kazura, Guiyun Yan y Guofa Zhou

Resumen

Antecedentes: Etiopía es uno de los países africanos donde el *Plasmodium falciparum* y el *P. vivax* coexisten. El monitoreo y la evaluación del estado actual de la transmisión de la malaria es un componente importante del control de esta enfermedad, ya que es una medida del éxito de las intervenciones en curso y guía la planificación de los esfuerzos futuros de control y eliminación.

Texto principal: Evaluamos los cambios en la política de control de la malaria en Etiopía de 1990 a 2016 y revisamos la dinámica de casos confirmados y clínicos de especies de *Paludismo* y las muertes informadas para todas las edades y menos de cinco años desde 2001 hasta 2016. Se analizó la incidencia anual de parásitos en los distritos para caracterizar la estratificación de la transmisión

de la malaria implementada por el Ministerio de Salud. Descubrimos que Etiopía experimentó cambios importantes entre 2003 y 2005 y el posterior ajuste en el diagnóstico de malaria, el tratamiento y la política de control de vectores. Las intervenciones contra la malaria se han intensificado representadas por el aumento de mosquiteros tratados con insecticida (ITN, por sus siglas en inglés) y la vaporización residual de interiores (IRS, por sus siglas en inglés), la mejora de los servicios de salud y el diagnóstico mejorado de la malaria. Sin embargo, las coberturas de los ITN y la IRS a nivel nacional fueron bajas, con un 64% de cobertura de los ITN en 2016 y una IRS de 92.5% en el mismo año y solo se implementó en áreas propensas a epidemias de >2500 m de elevación. La tasa de incidencia de las manifestaciones clínicas de la malaria disminuyó de un promedio de 43.1 casos por cada 1000 habitantes anualmente entre 2001-2010 a 29.0 casos por cada 1000 habitantes anualmente entre 2011-2016 y las muertes por malaria disminuyeron de 2.1 muertes por cada 100 000 personas anualmente entre 2001-2010 a 1.1 muertes por cada 100 000 personas anualmente entre 2011 y 2016. Se redujo el mapa de transmisión de la malaria y la alta transmisión se limita principalmente al área fronteriza internacional occidental. Proporción de *P. falciparum* el paludismo se mantuvo prácticamente sin cambios entre 2000 y 2016, lo que indica que se necesitan esfuerzos adicionales para suprimir la transmisión.

Conclusiones: la morbilidad y mortalidad por malaria se ha reducido significativamente en Etiopía desde 2001, sin embargo, la incidencia de casos de malaria sigue siendo alta y hubo lagunas importantes entre la posesión de los ITN y el cumplimiento en áreas maláricas. Se necesitan esfuerzos adicionales para apuntar al área de alta transmisión del oeste de Etiopía para mantener los logros alcanzados hasta la fecha.

Translated from English version into Spanish by Reina X. Sanjurjo and Natalia Rossi, through

TRANSLATORS
WITHOUT BORDERS
